# Early life environment and snoring in adulthood

**DOI:** 10.1186/1465-9921-9-63

**Published:** 2008-08-22

**Authors:** Karl A Franklin, Christer Janson, Thórarinn Gíslason, Amund Gulsvik, Maria Gunnbjörnsdottir, Birger N Laerum, Eva Lindberg, Eva Norrman, Lennarth Nyström, Ernst Omenaas, Kjell Torén, Cecilie Svanes

**Affiliations:** 1 Department of Respiratory Medicine and Allergology, University Hospital, Umeå, Sweden; 2 National Respiratory Center, Department of Anesthesia and Intensive Care, Karolinska Institute, Danderyd Hospital, Stockholm, Sweden; 3 Department of Respiratory Medicine and Allergology, Uppsala University, Uppsala, Sweden; 4 Department of Pulmonary Medicine, Landspitali University Hospital, Reykjavik, Iceland; 5 Department of Thoracic Medicine, Haukeland University Hospital, Bergen, Norway; 6 Institute of Medicine, University of Bergen, Norway; 7 Department of Public Health and Clinical Medicine, Division of Epidemiology and Public Health Sciences, Umeå University, Sweden; 8 Center for Clinical Research, Haukeland University Hospital, Bergen, Norway; 9 Department of Occupational and Environmental Medicine and Allergology, Sahlgrenska University Hospital, Göteborg, Sweden

## Abstract

**Background:**

To our knowledge, no studies of the possible association of early life environment with snoring in adulthood have been published. We aimed to investigate whether early life environment is associated with snoring later in life.

**Methods:**

A questionnaire including snoring frequency in adulthood and environmental factors in early life was obtained from 16,190 randomly selected men and women, aged 25–54 years, in Sweden, Norway, Iceland, Denmark and Estonia (response rate 74%).

**Results:**

A total of 15,556 subjects answered the questions on snoring. Habitual snoring, defined as loud and disturbing snoring at least 3 nights a week, was reported by 18%. Being hospitalized for a respiratory infection before the age of two years (adjusted odds ratio (OR) = 1.27; 95% confidence interval (CI) 1.01–1.59), suffering from recurrent otitis as a child (OR = 1.18; 95%CI 1.05–1.33), growing up in a large family (OR = 1.04; 95%CI 1.002–1.07) and being exposed to a dog at home as a newborn (OR = 1.26; 95%CI 1.12–1.42) were independently related to snoring later in life and independent of a number of possible confounders in adulthood. The same childhood environmental factors except household size were also related with snoring and daytime sleepiness combined.

**Conclusion:**

The predisposition for adult snoring may be partly established early in life. Having had severe airway infections or recurrent otitis in childhood, being exposed to a dog as a newborn and growing up in a large family are environmental factors associated with snoring in adulthood.

## Background

About 16% of middle-aged men and 7% of women snore habitually [1,2]. They suffer from daytime sleepiness and run an increased risk of cardiovascular diseases [3-6]. Snoring is a sign of increased upper airway resistance, usually due to a compromised upper airway during sleep. Snoring and daytime sleepiness are also symptoms of obstructive sleep apnea.

Enlargement of the lymphatic system with hypertrophy of the tonsils and the tongue are common causes of a reduction in the size of the upper airways. Infants snore during respiratory infections, and school children snore and suffer from sleep apnea when their tonsils are enlarged [7-10]. Mandibular retrognathia and narrowing of the lateral pharyngeal area reduce the upper airway size, with snoring and sleep apnea as a result in adults [11-13]. Obesity, age, smoking and chronic bronchitis are other risk factors for snoring among adults [14,15].

There has been great attention to research focusing on the early life origins of adult disease during the last two decades [16]. Increasing evidence show that early life environment may influence health throughout life. Risk factors for adult cardiovascular diseases and diabetes mellitus for example, include maternal smoking, low birth weight and socio-economic class [17-19]. Exposure to pets and growing up on a farm appear to be protective for allergy, while severe respiratory infections in childhood and living in a large family increase the risk of asthma [20-23].

To our knowledge there are no studies investigating whether the susceptibility to adult snoring and sleep apnea could be partly determined by early life environment. In the present paper we aimed to investigate whether environmental factors in childhood are associated with snoring later in life.

## Methods

A postal questionnaire was sent to 21,802 men and women aged 25–54 years in 1999–2001, with two reminders to non-responders [14]. Altogether 16,190 subjects (74%) responded. The responders were more likely being women (52.9 vs. 47.7%, p < 0.001) and slightly older (40 ± 7 vs. 39 ± 7 years, p < 0.001) than the non-responder. This sample was enrolled in the Respiratory Health in Northern Europe (RHINE) survey, which is a follow-up of subjects who participated in the European Community Respiratory Health Survey (ECHRS) in 1990–1994 [24]. The subjects were randomly selected from population registers in Reykjavik in Iceland, Bergen in Norway, Umeå, Uppsala and Göteborg in Sweden, Aarhus in Denmark and Tartu in Estonia. Ethics committees in Aarhus, Bergen, Göteborg, Reykjavik, Tartu, Umeå and Uppsala approved the study protocol. All the subjects gave their written informed consent for participation. The full protocols are available on the internet [25,26].

### Questions on snoring and daytime sleepiness

Loud and disturbing snoring, and daytime sleepiness during the last few months was assessed using a five-point scale according to the Basic Nordic Sleep Questionnaire: never, less than once a week, 1–2 days or nights a week, 3–5 days or nights a week and almost every day or night [27]. Habitual snoring was defined as loud and disturbing snoring at least three nights a week. A total of 15,556 subjects answered the questions on snoring. Daytime sleepiness was defined as feeling sleepy during the daytime at least one to two days a week

### Questions on childhood environment

The following questions concerning early life were included in the questionnaire:

"How old was your mother when you were born?"

"Was there any pet in your home at the time when you were born?" Alternative responses: Dog, cat, other pet.

"Was there any pet in your home when you were a child?" Alternative responses: Dog, cat, other pet.

"Were you hospitalized for a respiratory infection at any time before the age of 2 years?" Alternative responses: yes or no.

"Did you suffer from recurrent otitis in childhood?" Alternative responses: yes or no.

"What education did your mother have?" "What education did your father have?" Alternative responses: primary school, high school, university, other.

"How many people lived in your home, when you were five years old?"

In addition, questions on maternal smoking history during pregnancy and when the subjects were younger than 5 years of age were included in the questionnaire used in Bergen, Norway.

### Status in adulthood

Asthma was defined as answering yes to the question: "Have you had an attack of asthma in the last 12 months?" and/or "Are you currently taking any medicine (including inhalers, aerosols or tablets) for asthma?"

Allergic rhinitis was defined as answering yes to the question: "Do you have any nasal allergies including hay fever?"

Chronic bronchitis was defined as a negative answer to: "Have you ever had asthma?" and positive answers to all the following three questions: "Do you usually bring up phlegm or do you have phlegm, which you have difficulty bringing up?", "Do you bring up phlegm in this way almost every day for at least three months every year?" and "Have you had episodes of this kind for at least two years in a row?".

A pack-year of smoking corresponded to 20 cigarettes a day/year. Body mass index (BMI) in kg/m^2 ^was calculated from self-reported height and weight. Type of dwelling was used as a proxy for socio-economic status.

### Statistical methods

Chi-square test was used to test for differences between proportions and the Mann-Whitney U-test was used to test for differences between continuous variables. The associations between early life factors and adult snoring were analyzed with multivariable logistic regression in one model, adjusting for childhood environmental factors and possible confounders in adulthood such as chronic bronchitis, asthma, allergic rhinitis, smoking, BMI, age, gender, type of dwelling and centre. In these models we included variables that were related to habitual snoring with a p-value < 0.1 in univariate analysis.

Interactions by gender and BMI were tested for early life factors significantly associated with snoring (p < 0.05) in the adjusted model. Potential heterogeneity between centers was addressed by meta-analysis [28]. Data are presented as odds ratio (OR) and 95% confidence interval (CI). The adjusted proportion of snoring that could be explained by different risk factors was calculated as the population attributable fraction (PAF). All analysis were performed using Stata 8.0.

## Results

The characteristics of the study population according to centre are presented in Table 1. Habitual snoring was reported by 2,851 subjects (18%). Habitual snorers were more often men, more obese, older, had a higher prevalence of asthma and chronic bronchitis and had smoked more than non-snorers (Table 2). The prevalence of exposure to different environmental factors in early life among snorers and non-snorers is given in Table 3. Snoring subjects came from homes with lower parental education, larger household size and more pets when newborn, they had more often been hospitalized for respiratory infection before the age of 2 years, and they had more often had recurrent otitis in childhood (Table 3).

**Table 1:** Characteristics of the population.

	Reykjavik (n = 1,969)	Bergen (n = 2,506)	Umeå (n = 2,640)	Uppsala (n = 2,572)	Göteborg (n = 2,188)	Aarhus (n = 2,607)	Tartu (n = 1,708)	All subjects (n = 16,190)
Women (%)	54.6	51.9	51.5	52.5	54.2	52.2	56.1	52.9
Age (years)	41 ± 7	41 ± 7	41 ± 7	40 ± 7	40 ± 7	39 ± 7	36 ± 7	40 ± 7
BMI (kg/m^2^)	25.3 ± 4.0	24.7 ± 4.0	25.2 ± 3.9	24.6 ± 3.9	25.0 ± 3.9	24.3 ± 4.2	24.2 ± 4.2	24.8 ± 4.1
Habitual snoring (%)	20.6	16.9	20.7	18.6	20.4	17.7	12.0	18.3

BMI = body mass index. Means are expressed ± SD.

**Table 2:** Characteristics of habitual and non-habitual snorers in adulthood.

	Snorers n = 2,851	Non-snorers n = 12,705	p-value
Women n (%)	956 (34)	7,267 (57)	< 0.001
Age (years)	42 ± 7	39 ± 7	<0.001
BMI (kg/m^2^)	27 ± 5	24 ± 4	< 0.001
Smoking (pack-years)	7.1 ± 11.5	3.8 ± 7.7	< 0.001
Asthma n (%)	256 (9.0)	774 (6.1)	< 0.001
Allergic rhinitis n (%)	686 (25)	2,875 (23)	0.07
Chronic bronchitis n (%)	299 (11)	531 (4.2)	< 0.001

BMI = body mass index. Means are expressed ± SD.

**Table 3:** Early life characteristics according to adult habitual snoring

	Snorers n = 2,851	Non-snorers n = 12,705	p-value
Hospitalized for respiratory infection before 2 years of age n (%)	132 (4.7)	479 (3.8)	0.03
Otitis in childhood n (%)	617 (22)	2,490 (20)	0.006
Dog at home when newborn n (%)	617 (22)	2,173 (17)	< 0.001
Dog at home in childhood n (%)	1,156 (41)	4,890 (39)	0.033
Cat at home when newborn n (%)	565 (24)	2,095 (19)	< 0.001
Cat at home in childhood n (%)	1,036 (43)	4,690 (43)	0.779
Other pet at home when newborn n (%)	164 (6.8)	533 (4.9)	< 0.001
Other pet at home in childhood n (%)	356 (15)	1,557 (14)	0.435
Household size > 5 n (%)	673 (24)	2,529 (20)	< 0.001
Mother's age at delivery (years)	27.8 ± 6.3	28.1 ± 6.1	0.096
Mother university educated n (%)	205 (7.4)	1,376 (11)	< 0.001
Father university educated n (%)	373 (14)	2,146 (17)	< 0.001

Means are expressed ± SD.

The associations of childhood factors with adult snoring when adjusting for potential confounding factors are presented in table 4. Being hospitalized for a respiratory infection before the age of two years, suffering from recurrent otitis as a child, being born by a younger mother, growing up in a large family and being exposed to a dog at home as a newborn were significantly associated with adult snoring, independent of childhood exposure to cats or other pets, parents' education, adult chronic bronchitis, asthma, allergic rhinitis, active smoking, BMI, age, gender, current type of dwelling and centre (Table 4). The same childhood factors except family size were also associated with snoring accompanied by daytime sleepiness (Table 4).

**Table 4:** Adjusted odds ratios* for the associations between early life factors and adult habitual snoring, and habitual snoring with daytime sleepiness combined (n = 13,484).

	Habitual snoring		Habitual snoring with daytime sleepiness	
	Unadjusted OR (95% CI)	Adjusted OR (95% CI)	Unadjusted OR (95% CI)	Adjusted OR (95% CI)
Hospitalized for respiratory infection before 2 years of age	1.27 (1.04–1.54)	1.27 (1.01–1.59)	1.46 (1.16–1.83)	1.40 (1.07–1.81)
Otitis in childhood	1.15 (1.04–1.27)	1.18 (1.05–1.33)	1.37 (1.22–1.56)	1.34 (1.18–1.54)
Dog at home when newborn	1.34 (1.21–1.48)	1.26 (1.12–1.42)	1.38 (1.22–1.56)	1.35 (1.17–1.54)
Cat at home when newborn	1.30 (1.17–1.44)	0.99 (0.86–1.14)	1.29 (1.13–1.47)	1.01 (0.85–1.20)
Other pet at home when newborn	1.43 (1.19–1.71)	1.13 (0.90–1.42)	1.51 (1.21–1.87)	1.20 (0.92–1.56)
Household size (one more person)	1.07 (1.04–1.10)	1.04 (1.002–1.07)	1.05 (1.02–1.09)	1.03 (0.99–1.07)
Mother's age at delivery (per 5 years' increase)	0.98 (0.95–1.01)	0.96 (0.93–0.999)	0.96 (0.92–0.997)	0.95 (0.90–0.99)
Asthma	1.52 (1.31–1.77)	1.14 (0.94–1.37)	1.85 (1.56–2.19)	1.28 (1.03–1.57)
Allergic rhinitis	1.09 (0.99–1.20)	1.22 (1.08–1.36)	1.31 (1.16–1.47)	1.38 (1.20–1.58)
Chronic bronchitis	2.72 (2.34–3.15)	2.33 (1.95–2.80)	3.34 (2.84–3.93)	2.76 (2.27–3.35)
Smoking (per 5 pack years increase)	1.20 (1.17–1.22)	1.15 (1.12–1.18)	1.18 (1.15–1.21)	1.13 (1.10–1.17)
Body mass index (per 5 kg/m^2 ^increase)	2.04 (1.94–2.15)	1.82 (1.72–1.93)	1.80 (1.70–1.90)	1.69 (1.58–1.80)

CI = confidence interval, *The logistic regression also included parents' education age, gender, type of dwelling and centre.

Stratifying by BMI, an association between hospitalization for respiratory infection before age 2 years and adult snoring was only observed among overweight subjects (6,401 subjects with BMI > 25 kg/m^2^). Among the overweight, early hospitalization for respiratory infection was associated with adult snoring with an OR = 1.67; 95% CI 1.26–2.20, while among subjects with normal weight the OR was 0.82; 95% CI 0.55–1.23. This difference in associations of childhood respiratory infections with adult shoring according to BMI was highly significant (p _interaction _= 0.006). There was no significant interaction by gender, and there was no heterogeneity between centers.

**Figure 1:**
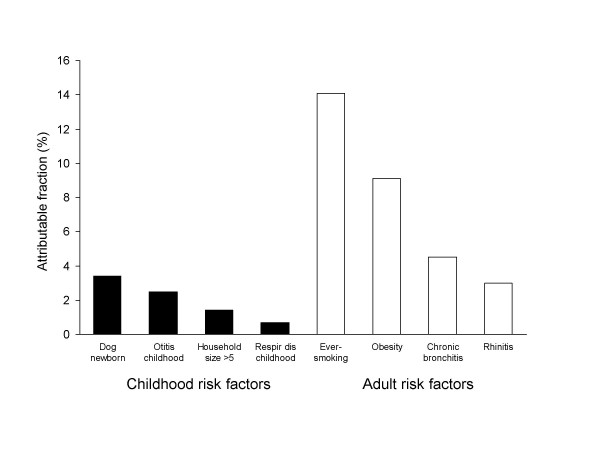
Adjusted population attributable fraction for childhood (black bars) and adult risk factors (white bars) for snoring.

The adjusted population attributable fraction for snoring of having been exposed to a dog when newborn was 3.4% while the corresponding figures were 2.5% for otitis, 1.4% for growing up in family of more than 5 persons and 0.7% for being hospitalized for a respiratory disease before the age of 2. Of the adult risk factors, the adjusted population attributable fraction for snoring was 3.0%, for rhinitis 4.5%, for chronic bronchitis 9.1%, for obesity (BMI ≥ 30 kg/m^2^) 14.1% and for ever smoking (Figure 1).

Eighteen percent of subjects from Bergen reported that their mother had smoked during pregnancy and 33% reported that she had smoked when they were younger than 5 years of age. Neither maternal smoking during pregnancy (adjusted OR = 1.06; 95% CI 0.63–1.79), nor maternal smoking in childhood (adjusted OR = 1.05; 95% CI 0.66–1.67) were related to adult snoring in this sub-sample (n = 2,506).

## Discussion

Being hospitalized for a respiratory infection before the age of two years, having had recurrent otitis in childhood, having been exposed to a dog as a newborn, having grown up in a large family were associated with habitual snoring later in life. These findings were independent of other childhood exposures and adult risk factors for snoring. When considering habitual snoring with daytime sleepiness combined, the same childhood factors were associated with increased adult risk. Our observations were demonstrated in a large population study in Northern Europe and were consistent across the seven centers. The findings are new and indicate that a predisposition for adult snoring and possibly also for obstructive sleep apnea could be established early in life.

Obesity is a major cause of snoring and sleep apnea. It is, however, important to increase knowledge about other preventable causes of habitual snoring, since a large number of snorers suffer from daytime sleepiness and an increased risk of cardiovascular disease and even early death [1,3-6,29]. The present study showed that early life environment may be of importance for snoring later in life. Further knowledge of this subject could contribute to primary prevention of adult snoring.

Our results indicate an association between early life environment and snoring later in life. It is, however, only possible to speculate about causal relationships and mechanisms based on the present data. Previous studies have shown that children with large tonsils develop retrognathia and posteriorly inclined mandibles as a result of changes in tongue posture and mouth breathing [30,31]. Studies on growing monkeys has also shown that induced oral respiration leads to a lowering of the chin, a steeper mandibular plane angle, and an increase in gonial angle as compared with control animals [32]. It is possible that subjects reporting otitis, severe respiratory infections or living in a large family in childhood more frequently had infections in the upper airways with hypertrophy of the tonsils and subsequent narrowing of the adult upper airways. Further, endotoxins are proinflammatory cell wall components from gram-negative bacteria and airborne endotoxins that are prevalent especially in homes with dogs [33]. We hypothesize that infections in childhood and exposure to airborne endotoxins in infancy stimulate the lymphatic system with subsequent enlargement of the tonsils. Remaining large tonsils or retrognathia due to large tonsils in childhood may compromise the upper airways, and could explain the associations between early life factors and snoring in adulthood as observed in this study. Unfortunately, we do not have information about history of tonsillectomy and/or adenoidectomy which might have been valuable for further understanding of these mechanisms.

A severe infection in childhood was only related to snoring later in life among overweight subjects, indicating that subjects who suffered from severe infections in childhood run a higher risk of habitual snoring if they become obese later in life. It is difficult to speculate on this relationship, but it seems reasonable that obesity, which is a common cause of snoring, increase potential negative consequences related to severe airways infections early in life.

The strengths of the present study are the large number of subjects, the multi-centre structure, the detailed analysis of childhood environmental factors and the high response rate to the questionnaire. The response rate analysis showed that men and younger subjects were slightly underrepresented. As the absolute differences between non-responders and responders were relatively small and we do not think that this has affected our results substantially.

The present analysis is limited by recall bias in assessments of childhood environment based on information in adulthood. A recent analysis, based on a multi-cultural study of childhood pet keeping, indicated that adults report important childhood events like having a dog or cat very consistently [34]. We therefore assume that the reporting of pets and household size in this study is fairly reliable. Reports of childhood hospitalization could possibly be biased with regard to childhood social class and subsequent respiratory infection; however, the analyses were adjusted for parental education. It is unlikely that the misclassification of any of these childhood factors would be differential with regard to adult snoring and we believe that the misclassification in this study is non-differential and may have attenuated the effects.

Other limitations include residual confounding from variables not included in the present study, such as current pet keeping, current household size, seasonal allergies, mouth breathing in sleep and childhood snoring. It is for example possible that persons exposed to pets during childhood are more likely to keep pets as adults, and that the association with current snoring and dogs is explained by current exposure rather than by previous exposure.

Snoring was based on subjective reports, which is a common limitation in epidemiological studies. Subjective reports are, however, the most commonly used instrument for measuring snoring, in part because of the technical problems involved with microphone recordings as well as the ability of subjective reports to give an average of the subject's degree of snoring, whereas the result of a single night's recording may be misleading. Objective recordings using microphones correlate well with subjective snoring in young adults [35].

## Conclusion

The predisposition for adult snoring and possibly also for obstructive sleep apnea may be partly established early in life. Having had severe airway infections or recurrent otitis in childhood, being exposure to a dog as a newborn, and growing up in a large family appear as possible risk factors for snoring in adulthood. We speculate that these factors may enhance inflammatory processes and thereby alter upper airway anatomy early in life causing an increased susceptibility for adult snoring. The presented findings are new and suggest that further knowledge about early life environment might contribute to primary prevention of snoring.

## Abbreviations

BMI:  Body mass index; CI: Confidence interval; OR: Odds ratio.

## Competing interests

The authors declare that they have no competing interests.

## Authors' contributions

KAF participated in the design, analysis, interpretation and drafted the manuscript. CJ participated in the design and coordination of the study, acquisition of data and to critically draft the manuscript. CS conceived the study, performed the analysis and helped to draft the manuscript. TG, AG, MG, BL, ENL, EN, LN, EO and KT participated in the design, acquisition of data and to critically draft the manuscript. All authors read and approved the final manuscript.
